# Tribute of the Board of Regents

**Published:** 1911-06

**Authors:** 


### Tribute of the Board of Regents

A meeting of the Board of Regents of the University of New York was held April 19, 1911. Reference was made by the commissioner of education, Dr. Andrew S. Draper, to the deaths of Drs. Macdonald, Ely and Potter, members of the State Board of Medical Examiners, and Regent Vander Veer was requested to present a suitable memorial to be inserted in the minutes.

The following is an abstract of the memorial to Dr. William Warren Potter:

William Warren Potter, president of the State Board of Medical Examiners, passed from this life at his home in Buffalo, N. Y., March 14, 1911.

During the years in which Dr. Potter has been connected with the State Board it is impossible to properly estimate the value of services so faithfully discharged as examiner in the department of obstetrics and gynecology. In the exacting duties that devolved upon him in this department he bestowed the most conscientious care of his great ability.

Dr. Potter was signally honored throughout his long and active career both by the members of the medical profession and by distinguished citizens of the State of New York. He served as surgeon of volunteers three years of the civil war and was brevetted by the President of the United States lieutenant colonel for faithful and meritorious service on the field of battle.

The literature of gynecology and obstetrics has been enriched, and the practice of this special department materially advanced, during the time of Dr. Potter's connection with the State Board of Medical Examiners.

